# Effects of external radiation in a co-culture model of endothelial cells and adipose-derived stem cells

**DOI:** 10.1186/1748-717X-8-66

**Published:** 2013-03-20

**Authors:** Frank Haubner, Michaela Leyh, Elisabeth Ohmann, Fabian Pohl, Lukas Prantl, Holger G Gassner

**Affiliations:** 1 Department of Otorhinolaryngology, Division of Facial Plastic Surgery, University of Regensburg, Regensburg, Germany; 2 Department of Radiotherapy, University of Regensburg, Regensburg, Germany; 3 Department of Plastic and Reconstructive Surgery, University of Regensburg, Regensburg, Germany

## Abstract

**Background:**

The inflammatory response clinically observed after radiation has been described to correlate with elevated expression of cytokines and adhesion molecules by endothelial cells. Therapeutic compensation for this microvascular compromise could be an important approach in the treatment of irradiated wounds. Clinical reports describe the potential of adipose-derived stem cells to enhance wound healing, but the underlying cellular mechanisms remain largely unclear.

**Methods:**

Human dermal microvascular endothelial cells (HDMEC) and human adipose-derived stem cells (ASC) were cultured in a co-culture setting and irradiated with sequential doses of 2 to 12 Gy. Cell count was determined 48 h after radiation using a semi-automated cell counting system. Levels of interleukin-6 (IL-6), basic fibroblast growth factor (FGF), intercellular adhesion molecule-1 (ICAM-1) and vascular cell adhesion molecule-1 (VCAM-1) were determined in the supernatants using enzyme-linked immunosorbent assay (ELISA). Irradiated HDMEC and ASC as well as non-irradiated co-cultures, HDMEC or ASC respectively were used as controls.

**Results:**

Cell count was significantly reduced in irradiated co-cultures of HDMEC and ASC compared to non-irradiated controls. Levels of IL-6, FGF, ICAM-1 and VCAM-1 in the supernatants of the co-cultures were significantly less affected by external radiation in comparison to HDMEC.

**Conclusion:**

The increased expression of cytokines and adhesion molecules by HDMEC after external radiation is mitigated in the co-culture setting with ASC. These in vitro changes seem to support the clinical observation that ASC may have a stabilizing effect when injected into irradiated wounds.

## Introduction

Wound healing is impaired after radiation therapy and severe peri- and postoperative complications may ensue [1,2]. The management of these complications after irradiation may result in unsatisfactory outcomes despite extensive reconstructive efforts. Therefore the research of novel therapeutic strategies to improve cutaneous wound healing after radiation continue to be of major interest.

Human mesenchymal stem cells have been utilized for the therapy of radiogenic ulcers. Adipose-derived stem cells (ASC) have been described for the therapy of limited local tissue injuries and seem to improve angiogenesis and the reconstitution of dermal architecture [3]. Zuk et al. documented that lipoaspirates contain multipotent cells and are an alternative stem cell source to the bone marrow-derived stem cells [4]. An important advantage of adipose-derived stem cells over other sources of stem cells is that they are easily obtained in large quantities by liposuction [5,6]. Also their potency to synthesize growth factors and cytokines shows promise for the use in skin repair and regeneration [7-9]. Promising results of ASC injections after radiation have been described anecdotally, but larger prospective clinical studies investigating the effect of adipose-derived stem cell injections on radiogenic wounds are not available [10]. Hadad et al. developed a wound healing model to study such effects in pigs [11]. They found no effect of ASC injections alone but they documented improved wound healing by a combination therapy of ASC and platelet rich plasma injections into irradiated wounds. The most important effects were accelerated wound closure and an increased microvessel density after the combined treatment. One important conclusion of this study was that improvement of microcirculation after radiation therapy may be the key to treating wound healing compromise.

An important contributor to compromised wound healing in this context is endothelial dysfunction, which manifests in atherosclerosis, fibrosis and vascular occlusion [12]. Endothelial cells display a high sensitivity to radiation injury, yet these cells play an essential role in the complex network of wound healing. As previously reported [13] we documented a pro-inflammatory cytokine release and elevated levels of adhesion molecules after external radiation of microvascular endothelial cells (HDMEC). The aim of the present study was to examine if these effects are altered when HDMEC and ASC are cultivated in co-culture.

## Material and methods

### Cell culture

Human dermal microvascular endothelial cells (HDMEC, adult donor, catalog number C-12212; PromoCell, Heidelberg, Germany) were maintained in endothelial cell growth medium MV (Promo-Cell, catalog number C-22020) and used for experiments at passages 5 through 6. Adipose-derived stem cells (ASC, isolated as described previously by Gehmert et. al [14].) were maintained in ASC medium (αMEM containing 20% FBS, 2 mM L-glutamine and, 1% penicillin/streptomycin, Sigma, St. Louis, MO, USA) and were used for experiments at passages 5 through 6.

Briefly, subcutaneous fat tissue - obtained from patients undergoing elective body-contouring procedures - was washed in phosphate-buffered saline, and minced into pieces of <2 mm^3^. Serum-free MEM (1 ml/1 g tissue) and LiberaseBlendzyme 3 (Roche Diagnostics, Basel, Switzerland) (2 U/1 g tissue) were added and incubated under continuous shaking at 37°C for 45 min. The digested tissue was sequentially filtered through 100- and 40-μm filters (Fisher Scientific, Schwerte, Germany) and centrifuged at 450 *g* for 10 min. The supernatant was discarded and pelleted cells were washed twice with Hanks’ balanced salt solution (Cellgro, Manassas, VA, USA). Plastic-adherent passage 0 cells were then grown in culture vials (Greiner Bio-one, Frickenhausen, Germany) followed by daily washes to remove red blood cells and non-attached cells. After cells reached 80% confluence in passage 0, they were seeded at a density of 3000 cells/cm2. The culture incubator was set at 37°C with 5% carbon dioxide. According to the literature ASC remain their differentiation capacity up to passage 15 [15]. ASC cultures were harvested and molecular characterized from the Applied Stem Cell Research Center of the University of Regensburg.

ASC isolation was in accordance with guidelines of the Declaration of Helsinki for biomedical research. Written consent was obtained from the Institutional Review Board (IRB) of the University of Regensburg to harvest ASC from patients (“Human mesenchymal stem cells as a target for development of cell based regenerative therapies”, IRB #08/117).

### Co-culture of ASC and HDMEC

As control HDMEC or ASC were seeded at a density of 70.000 cells per 6-well and were supplemented with 3 ml of respective culture medium. In the direct co-culture (1:2) 35.000 cells of each HDMEC and ASC were mixed and seeded in a 6-well and were supplemented with 3 ml endothelial cell growth medium. For the remaining culture time all cell types were supplemented with 3 ml HDMEC medium. After a cell adhesion time of 24 h medium was replaced.

### Cell-radiation

The 6-well plates were placed 48 h after seeding the cells on the acceleration treatment couch. 2 cm thick plates of perspex were positioned above and below the tissue culture flasks to compensate for the build-up effect, the irradiation was delivered via an anterior portal by a 6 MV linear accelerator (3 Gy/min; Primus, Siemens, Nuernberg, Germany) at room temperature as previously described by Pohl et al [16]. Dosimetric evaluations were performed to guarantee a homogenous dose distribution. The cells were irradiated with doses of 2Gy, 6Gy and 12Gy, respectively. Non-irradiated cells served as a control.

### Cell-harvesting

The co-culture supernatant was collected 48 h after irradiation, centrifuged 2 min at 13.000 rpm (14.196 g) and stored at −20°C for further analysis. HDMEC, ASC and co-cultures in the 6-wells were washed with PBS (phosphate-buffered saline, PAA laboratories, Pasching, Austria) and detached by incubation with 500 μl Trypsin/EDTA (Promo-Cell; catalog number C-41000) for 5 min at 37°C. Cell number was determined with the Cedex XS cell counter system (Innovatis, Basel, Switzerland). Cells were pelletized and stored at −20°C for further analysis.

### Enzyme-linked immunosorbent assay (ELISA)

Soluble cytokine production in the supernatants of co-cultures, ASC and HDMEC was tested by ELISA (DuoSet ELISA Development Systems; R&D Systems, Minneapolis, USA). Prior to use, the cell culture supernatants were centrifuged at 5000 rpm (2.100 g) for 5 minutes. The DuoSet kits human FGF basic (catalog No. DY233), human IL-6 (DY206), human ICAM-1 (DY720) and human VCAM-1 (DY809) were used according to the manufacturer’s instructions.

### Statistical analysis

Results are expressed as mean (SD) of at least three independent experiments (different donors) performed in triplicate. Statistical analysis was performed using the unpaired 2- tailed *t* test (SPSS for Windows; SPSS Inc, Chicago, Illinois, USA). Statistical significance was set at *P* < .05 (in figures marked with *). P values < .01 (**) and p values < .001 (***) were indicated respectively.

## Results

### Effect of irradiation on cell proliferation

**Figure 1:**
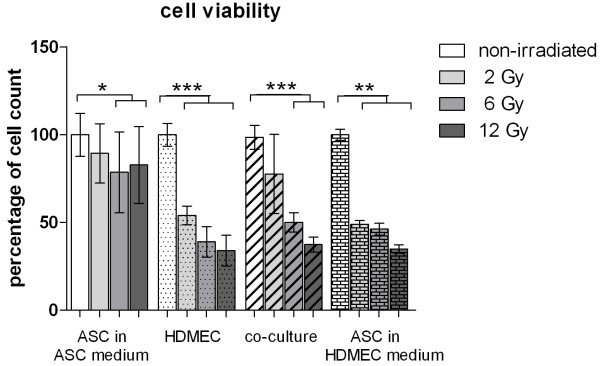
**Cell count of viable adipose-derived stem cells (ASC), human microvascular endothelial cells (HDMEC) and the co-culture of ASC and HDMEC 48 h after irradiation with 2 to 12 Gy compared to unirradiated control cells.** Error bars represent standard deviation. Statistical significance was set at p *< .*05 (*). P values < .01 (**) and p values < .001 (***) were indicated respectively.

In order to analyze the effect of irradiation on proliferation of ASC and the co-culture of ASC and HDMEC the number of viable cells 48 h after irradiation was determined using the Cedex XS Analyzer System as described in the “Material and Methods” section. Irradiation intensity of 2 Gy did not significantly reduce the cell number of ASC in ASC medium. Irradiation intensity of 6 and 12 Gy reduced the cell number significantly in comparison to non-irradiated control cells. External radiation of ASC in HDMEC medium resulted in a decrease of cell proliferation comparable to the effect of irradiation on HDMEC as shown previously (Figure 1).

Irradiation of HDMEC and ASC in a direct co-culture with intensities of 2 to 12 Gy caused a reduction in the cell number when compared to non-irradiated control cells (Figure 1).

Cell viability was observed via microscopic visualization and the cell counting system. Viability was consistently above 95% independent of irradiation status.

### Expression of cytokines, growth factors and adhesion molecules

Protein levels of cytokines, growth factors and adhesion molecules in the different cell culture supernatants 48 h after irradiation were analyzed using ELISA techniques.

The parameters ICAM-1 and VCAM-1 were not detectable in the supernatants of ASC in ASC medium.

**Figure 2:**
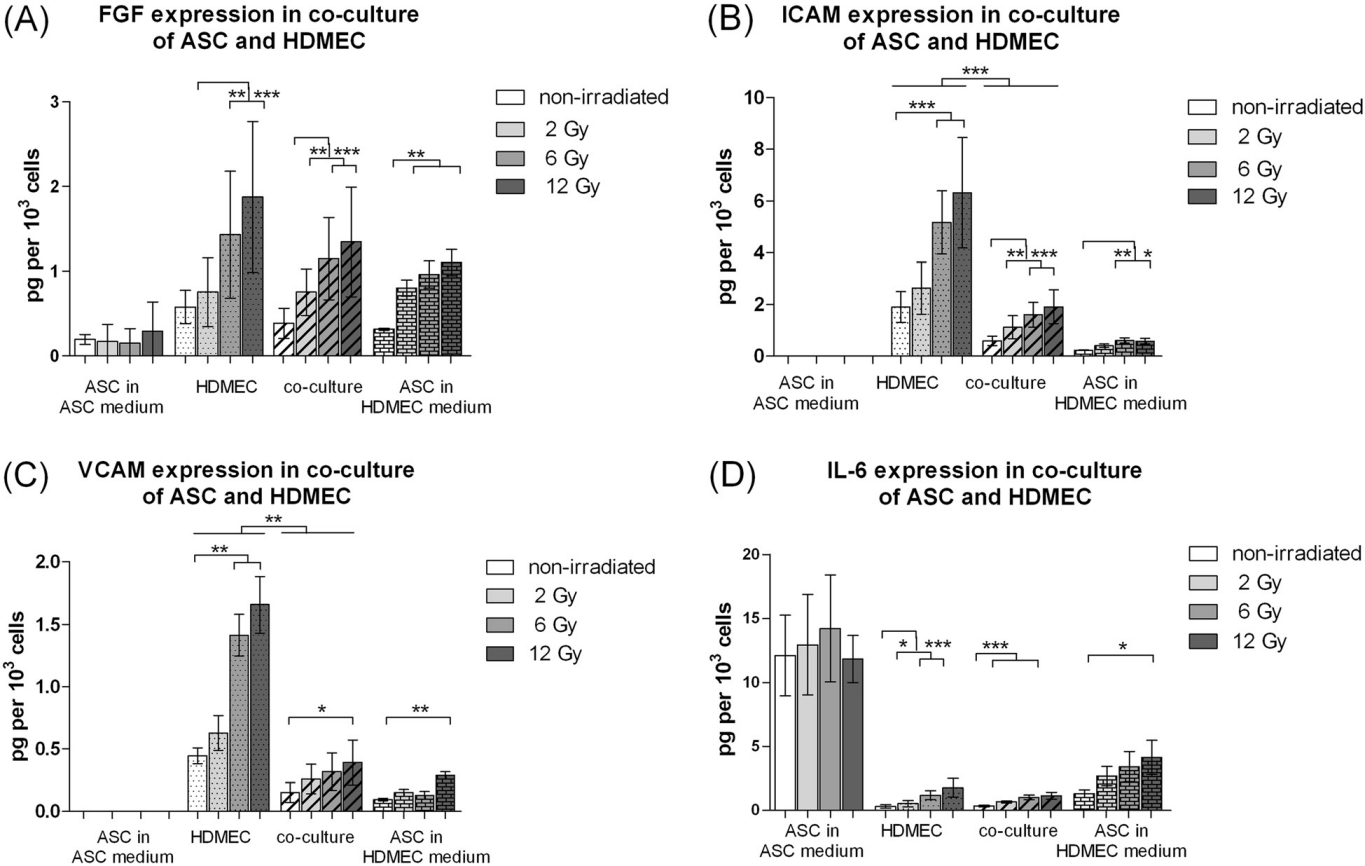
**Expression of bFGF (A), ICAM-1(B), VCAM-1(C) and IL-6 (D) 48 h after external radiation.** Protein levels in pg per 1 × 10^3^ cells in supernatant of ASC, HDMEC and the co-culture of HDMEC/ASC determined by ELISA. Error bars represent standard deviation (n = 4). Statistical significance was set at p *< .*05 (*). P values < .01 (**) and p values < .001 (***) were indicated respectively.

Human basic FGF was significantly increased 48 h after irradiation in the supernatants of HDMEC, ASC and the co-cultures of HDMEC-ASC using the HDMEC medium. Irradiation of ASC in ASC medium did not affect the FGF level in comparison to non-irradiated ASC (Figure 2A).

The soluble adhesion molecule ICAM-1 was significantly elevated after irradiation of HDMEC, ASC and the co-cultures of HDMEC-ASC in HDMEC medium (Figure 2B and C). The soluble adhesion molecule VCAM-1 was significantly elevated after irradiation of HDMEC (6 Gy and 12 Gy), ASC (12 Gy) and the co-cultures (12 Gy) of HDMEC-ASC in HDMEC medium (Figure 2B and C).

**Figure 3:**
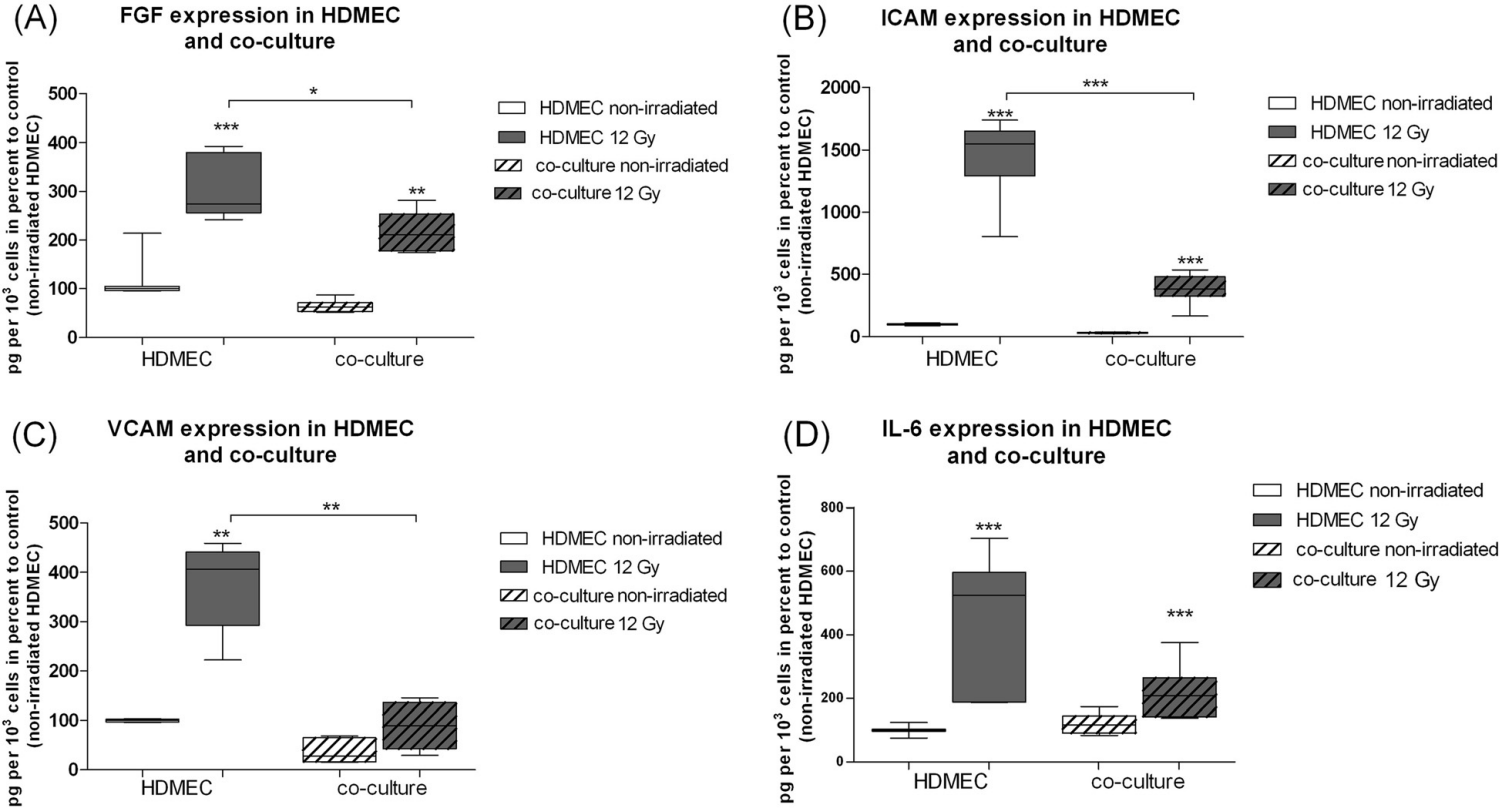
**Expression of bFGF (A), ICAM-1(B), VCAM-1(C) and IL-6 (D) 48 h after external radiation.** Protein levels in pg per 1 × 10^3^ cells in percent to non-irradiated HDMEC in supernatants of HDMEC and the co-culture of HDMEC-ASC determined by ELISA. Error bars represent standard deviation (n = 4). Statistical significance was set at p *< .*05 (*). P values < .01 (**) and p values < .001 (***) were indicated respectively.

There was a significant reduction of FGF concentration in the irradiated (12 Gy) co-culture in comparison to irradiated (12 Gy) HDMEC (Figure 3A).

The soluble adhesion molecule ICAM-1 was significantly elevated after irradiation of HDMEC, ASC and co-cultures. However, the ICAM-1 level was significantly reduced in HDMEC co-cultured with ASC in comparison to HDMEC.

The soluble adhesion molecule VCAM-1 was significantly elevated after irradiation of HDMEC, ASC and co-cultures. However, the VCAM-1 level was significantly reduced in HDMEC co-cultured with ASC in comparison to HDMEC 48 h after external radiation (Figure 3B and C).

Levels of IL-6 in HDMEC, ASC and the co-culture supernatants in HDMEC medium were significantly elevated after external radiation (Figure 2D).

Levels of IL-6 in ASC, HDMEC and co-culture in HDMEC medium were significantly elevated after external radiation. Under co-culture conditions of HDMEC and ASC there was a trend to lower IL-6 concentration in irradiated (12 Gy) co-cultures in comparison to irradiated (12 Gy) HDMEC (Figure 3D).

With respect to the absolute protein levels of 1 × 10^3^ cells there was a statistically significant decrease (p = 0,0175) of FGF from 1,88 pg +/− 0,89 pg to 1,36 pg +/− 0,65 pg in the irradiated HDMEC compared to irradiated co-cultures. Setting the initial protein synthesis of non-irradiated HDMEC to 100% there was a reduction of increase from 320% +/− 69% in irradiated HDMEC (12 Gy) to 229% +/− 46% in irradiated co-culture (12 Gy) (Figure 3A).

The absolute value of soluble ICAM-1 per 1 × 10^3^ cells was significantly reduced from 6,32 pg +/− 2,14 pg to 1,92 pg +/−0,65 pg in the irradiated (12 Gy) HDMEC compared to irradiated (12 Gy) co-cultures (p_ICAM_ = 0,0003). The absolute values of soluble VCAM-1 was significantly reduced from 1,66 pg +/− 0,22 pg to 0,39 pg +/− 0,18 pg per 1 × 10^3^ cells, in the irradiated (12Gy) HDMEC compared to irradiated co-cultures (12Gy) (p_VCAM_ = 0,0022). Relatively to non-irradiated HDMEC (100%) the reduction rate was significantly reduced for ICAM-1 from 1517% +/− 182% in irradiated (12Gy) HDMEC to 429% +/− 76% in irradiated (12 Gy) co-cultures. A significant reduction of VCAM-1 was observed in irradiated HDMEC: From 374% +/− 90% to 89% +/− 48% in irradiated co-culture (Figure 3B and C).

There was a non-significant trend to reduced amounts of IL-6 (p = 0,078) in the supernatants of the irradiated (12Gy) HDMEC compared to irradiated co-cultures (12Gy) (1,77 pg +/− 0,74 pg to 1,15 pg +/− 0,27 pg). The relative reduction due to the co-culture was 394% +/− 234% in irradiated HDMEC to 242% +/− 107% in irradiated co-cultures in comparison to non-irradiated HDMEC (100%).

## Discussion

Impairment of microcirculation and pro-inflammatory tissue response are key events in compromised wound healing [17].

The concept of therapeutic angiogenesis has become widely accepted in recent years [14]. Adipose derived stem cells are able to migrate and differentiate into endothelial cells. These cells are suggested to support angiogenesis in vitro [14]. Investigations of the potential role these cells may play in the context of compromised wound healing appear therefore justified.

Akita et al. described the use of ASC in a patient suffering from a radiogenic wound 40 years after irradiation. They used ASC-impregnated artificial dermis to cover the wound bed and injected ASC into the wound margins. The authors documented favorable results of wound healing and durable tissue regeneration after 1.5 years [10]. A pro-angiogenetic effect of stem cell injections was suggested by the authors, but other mechanisms may also serve to explain the findings in this study. Angiogenesis and optimizing of tissue microcirculation are suggested effects of stem cell injections in cutaneous wound healing. However a possible increase in the risk of tumor recurrence after stem cell therapy must remain an important consideration. Emerging evidence suggest that stem cells are also involved in tumor angiogenesis [18].

The aim of the present study was to focus on possible supportive effects of ASC in a co-culture setting with respect to important markers of microcirculation and inflammation. We decided to integrate human dermal microvascular endothelial cells into the co-culture experiments because of their important role in cutaneous wound healing. Endothelial cells display a high sensitivity to radiation injury. Therefore cellular events associated with an impaired function of endothelial cells are of particular clinical relevance.

Our previous cell culture study using a static HDMEC model documented a dose dependent up-regulation of pro-atherogenic adhesion molecules (ICAM-1, VCAM-1), which are associated with the endothelial dysfunction [13]. The present study reveals a dose dependent increase of both adhesion molecules after radiation of ASC and co-cultures of HDMEC-ASC in endothelial cell medium. The observation that ASC express adhesion molecules in endothelial cell medium only and not in stem cell medium suggest induction of ASC into endothelial cells by the medium. The capacity of ASC to differentiate into endothelial cells has already been described by Gehmert et al [14]. Irradiation of ASC in HDMEC medium resulted in an increase of the soluble adhesion molecule levels comparable to the effect of radiation on HDMEC mono-cultures. ASC medium (αMEM containing 20% FBS) was used in several studies in the past [14,19]. For the co-culture of ASC and endothelial cells it is essential to use medium with higher amounts of serum because HDMEC show an impaired growth behavior under-serum free conditions.

Interestingly the increase of expression of ICAM-1 and VCAM-1 was significantly lower in the co-cultures. One conclusion might be that ASC compensate the endothelial dysfunction after radiation by balancing the expression of soluble adhesion molecules. These observations are consistent with the profile of secreted cytokines and confirm recent studies concerning the supportive capability of hematopoiesis of ASC [20].

Proliferation and granulation tissue formation are regulated by the basic fibroblast growth factor (bFGF) [21]. This growth factor was elevated after external radiation in HDMEC as previously reported. The data of our present study revealed an increase of bFGF in ASC and co-cultures of HDMEC-ASC. The relative increase due to radiation was significantly lower in the co-culture environment. Up-regulation of bFGF is suggested to have positive effects on cutaneous wound healing. On the other hand, over-expression of bFGF may result in uncontrolled formation of granulation tissue [21]. Comparable co-culture experiments could not be identified in the literature but a positive effect of ASC injections was observed in an animal study by Forcheron [22]. These authors injected autologous adipose-derived stem cells into the skin of pigs irradiated with 50Gy. The authors observed improved clinical wound healing and an enhanced re-epitheliasation in the animals injected with adipose-derived stem cells. Previous studies suggest that porcine skin reacts similar to human skin to radiogenic injuries and wound healing mechanisms are similar in humans and pigs [22]. Also a cell culture study by Lee et al. supports the suggested stimulatory effects of adipose-derived stem cells on cutaneous wound healing [23]. In their study the proliferation of fibroblasts and their collagen synthesis was increased by conditioned medium of adipose-derived stem cells in vitro. Additional antioxidant effects of adipose-derived stem cells are reported by Kim et al. [24]. These effects were suggested to be mediated through the activation of dermal fibroblasts and keratinocytes via paracrine mechanisms.

External radiation of HDMEC evoked an increased expression of the pro-inflammatory cytokine IL-6 in our previous study. ASC and co-cultures of HDMEC-ASC showed a dose dependent increase of IL-6 in the supernatants. Previous studies documented high levels of IL-6 in ASC monocultures [25]. The present study confirms this finding of elevated IL-6 levels in cultures of ASC in the stem cell specific medium. This effect was mitigated under co-culture conditions. The decrease of IL-6 expression per cell after 12 Gy irradiation is due to the slightly increased cell count in this analysis. Possible changes in the cell cycle which would explain this finding have to be analyzed in the future.

Similar to the effects on bFGF, ICAM-1 and VCAM-1, the relative expression of IL-6 was also reduced comparing HDMEC and co-cultures. Previous cell culture studies documented an inflammatory response of endothelial cells to radiotherapy [26,27]. Compensation of this effect by injections of ASC could be promising in the treatment of radiogenic wounds.

With respect to the cell growth we found a negative correlation of cell count and radiation dose in the model of HDMEC and the co-culture model integrating ASC. Similar results were documented by Henning et al. [28], who used a cell culture model of human endothelial cells and aortic smooth muscle cells with irradiation doses up to 16 Gy. Interestingly external radiation of ASC mono-cultures in the stem cell medium did not significantly reduce the absolute number of viable stem cells. There was a marginally decreased cell count for irradiation with 6 Gy and 12 Gy with respect to the unirradiated controls, but this effect was not evident under co-culture conditions. In contrast, a dose dependent reduction of cell count was documented in ASC cultured with HDMEC medium. This effect was comparable to the effect observed after irradiation of HDMEC. This observation may further support the notion that ASC differentiate to endothelial cells in HDMEC medium. However, further analysis to clarify the differentiation to endothelial cells in the HDMEC medium and the interaction mechanism between the different cells in co-culture are necessary.

In consideration of these limitations, we feel the following conclusions are valid: The increased expression of cytokines and adhesion molecules by HDMEC after external radiation is mitigated in the co-culture setting with ASC. These in vitro changes seem to support the clinical observation that ASC may have a stabilizing effect when injected into irradiated wounds.

## Competing interest

Neither of the authors has an interest in any products, devices, instruments, or contracts that are related to this research.

## Authors’ contributions

LP and HG had the idea for the co-culture experiments. FH drafted the manuscript. EO and ML performed the experiments and prepared the figures. FP supported the manuscript by his radio-oncological knowledge. LP contributed by harvesting ASC for the experiments. All authors read and approved the final manuscript.
